# Impact of the Stress Status of Employees on the Enterprise Technology Management Cost Through Matter-Element Analysis Under Psychological Health Education

**DOI:** 10.3389/fpsyg.2021.593813

**Published:** 2021-07-16

**Authors:** Ximeng Zhang, Fanshen Han, Ming Gao, Lu Liu, Xiaping Wang

**Affiliations:** ^1^International Education College, Henan University of Science and Technology, Luoyang, China; ^2^Graduate School, Gachon University, Seongnam, South Korea; ^3^School of Economics and Management, University of Science and Technology Beijing, Beijing, China; ^4^School of Law, Chongqing University, Chongqing, China

**Keywords:** employee stress source, stress-coping strategies, matter-element analysis, development of mechanical artificial intelligence, mental health education

## Abstract

In this study, in order to analyze the stress sources and stress-coping strategies of employees in construction enterprises, to explore the influencing factors of enterprise technical management cost, and to offer suggestions for mental health education of employees, 372 employees of Shandong Construction Engineering Group Co., Ltd. were selected for a questionnaire survey. The influences of stress sources and stress-coping strategies on the mental health of employees were compared, based on different demographic variables. The evaluation model was constructed using the matter-element analysis to rank the factors influencing the enterprise technology management cost. The results showed that the stress value of work characteristics was the highest (4.26 ± 0.511), followed by the organizational structure and atmosphere (4.15 ± 0.382); stress-coping strategies at the individual level (1.84 ± 0.315) scored higher than that at the organizational level (1.67 ± 0.248) (*P* < 0.05). Notable differences were observed in balance between work and family between males and females (*P* < 0.05); in work characteristics, role orientation, personal relationship, and balance between work and family between subjects of different ages (*P* < 0.05); in work characteristics, and balance between work and family between the married and the unmarried (*P* < 0.05); and in role stress and work characteristics between subjects in different positions (*P* < 0.05). The evaluation results revealed that the factors influencing the technology management cost of enterprises included price index, development cost, fixed investment proportion, power equipment rate, mechanical artificial intelligence, labor cost, rate of technical equipment, the output value, informatization of technology management, and national policy. In conclusion, the two major sources of stress for employees in Luoyang Construction Engineering Group Co., Ltd. were as follows: (1) work characteristics and (2) organizational structure and atmosphere. Besides, many employees adopted the stress-coping strategies at the individual level, and enterprises needed to strengthen the psychological health education for employees at the organizational level. In practice, the enterprise needed to add importance to the development of mechanical artificial intelligence, informatization of technology management, and national policy.

## Introduction

Stress is a very common mental health phenomenon in the present society, which refers to the physical and mental stress response of people, caused by external environmental factors (Waters et al., 2020). With the acceleration of the integration of global economy, the competition among enterprises is intensifying, and the fast-paced production eventually increases the pressure on employees, resulting in various mental health problems (O’Halloran et al., 2020; Rodríguez et al., 2020). Proper stress is naturally beneficial and can motivate individuals to work harder, whereas excessive and long-term stress will negatively affect physical and mental health of employees, and even cause a series of group problems, such as slacking, which prevents enterprises from operating normally (Dohi et al., 2019; Lambert et al., 2019; Rahman et al., 2019). Only in the United States, more than US$150 billion a year is lost as a result of frequent absenteeism, absent-mindedness, and reduced creativity. Unfortunately, in practice, people focus on refining production skills at the individual and organizational levels, but almost ignore mental health and occupational stress of employees (Robinson et al., 2019). In addition, the service institutions specializing in mental health education of enterprise employees have not been truly popularized in China. As a result, it is impossible to systematically analyze the stress of enterprise employees (Sekhon et al., 2019; Watson and Chinnadurai, 2019). Hence, the stress of employees was discussed in the study, so as to provide a theoretical basis to carry out mental health education and alleviate stress of employees.

Enterprise technology management is a subsystem of the entire enterprise management system. It is the general term for a series of management activities, concerning enterprise technology development, product development, technological transformation, technological cooperation, and technology transfer (Davis and Comeau, 2020; Jnr et al., 2020). It aims to establish scientific work procedures, which is in accordance with the law of scientific and technological work, so as to reasonably use technologies and resources, and transform them into realistic productivity, thereby raising economic benefits (Saeidi et al., 2019). As economy is progressed continuously, the cost of enterprise technology management is also increasing year by year. For construction enterprises, the technical management cost refers to the cost to implement technical plans, material plans, and other preparation plans (Kanaan et al., 2019; Mayer et al., 2019). The matter-element analysis is a method to solve contradictory problems, as proposed by Chinese scholar Cai Wen in the 1980s. As an interdisciplinary subject of thinking science, system science, and mathematics (Zabukovšek et al., 2019), it can solve incompatible problems. The matter-element analysis can rank and sort the factors influencing technical management cost of construction enterprises, so as to control technical management cost effectively (Tribst et al., 2020). This study adopted the matter-element analysis to explore the related factors influencing technology management cost of construction enterprises.

In addition, the occupational stress is common, and it is urgent to increase the cost of enterprise technology management. Based on this, 372 employees of Luoyang construction enterprise were selected as the research subjects, and a questionnaire survey was conducted, to analyze the differences in stress sources and stress-coping strategies between various demographic variables. The matter-element analysis method was used to construct an evaluation model, to comprehensively evaluate the factors influencing the stress of employees and the technology management cost of construction enterprises.

## Methodology

### Research Subjects

In this study, 372 employees of Shandong Construction Engineering Group Co., Ltd. were selected as the research subjects. The average age was 33.51 ± 11.65 years, ranging from 21 to 47 years. Five hundred and eighty-two questionnaires were distributed, with 517 recovered. After the questionnaires with invalid data and incomplete questions were removed, there were 392 left, and the recovery rate was 84.54%.

Table 1 shows the demographic variables of the subjects. In terms of gender, the ratio of male-to-female employees was about 7:3, which was in line with the actual situation of more men than women in the construction industry. In terms of age, employees aged between 30 and 40 years accounted for the biggest portion (40.27%), while employees aged between 21 and 30 years accounted for the smallest portion (27.98%). This reflected the current situation in construction enterprises, that is, obvious ideological fluctuation of young people and unstable structure of technical team. In terms of educational background, the proportion of employees with bachelor’s degree was the largest (47.72%). In terms of work positions, ordinary technical personnel accounted for 71.89%. In terms of marital status, the married accounted for 64.28%.

**TABLE 1:** Basic information of employees of the tested enterprise.

Variables	Classification	Number of the samples	Proportion
Age	21–30	104	27.98%
	30–40	150	40.27%
	40–47	118	31.75%
Educational background	Junior college or below	63	17.02%
	Bachelor’s degree	178	47.72%
	Master’s degree and above	131	35.26%
Work position	General technician	267	71.89%
	Supervisory engineering staff	105	28.11%
Gender	Male	254	68.39%
	Female	118	31.61%
Marriage	Married	239	64.28%
	Unmarried	133	35.72%

### Factors Influencing Technical Management Cost of Enterprises

As shown in Table 2, based on the earlier literature, structured interviews were conducted, with 20 personnel with rich technical management experience in construction enterprise involved. Finally, 20 influencing factors were selected.

**TABLE 2:** The influencing factors.

Number	Influencing factor	Number	Influencing factor
1	Location of the enterprise	11	Labor cost ratio
2	Enterprise scale	12	Technical equipment rate
3	Price index	13	Economic globalization
4	Development cost ratio	14	Working population
5	Fixed investment proportion of enterprises	15	Enterprise production value
6	Enterprise labor productivity	16	Employee structure
7	Power equipment rate	17	Profit rate of construction industry
8	Contracting mode	18	Technical management informatization
9	Mechanization and artificial intelligence development	19	Corporate culture
10	Number of construction enterprises	20	National policy

### Evaluation Model of the Factors Influencing the Technology Management Cost of Enterprises

Based on the summary of influencing factors in Table 2, the matter-element analysis (Song et al., 2019) was used to construct the evaluation model of influencing factors of enterprise technical management cost. The thing was set as *N*, the characteristic of *N* was *c*, the corresponding value *c* was set as *v*, and *R* = (*N*,*c*,*v*) was the basic unit that described *N*. The influencing factors of technical management cost of 20 construction enterprises were set as *n* characteristics, and the matter-element matrix was established as follows:

(1) $$R=\left(N,c,v\right)=\left[\begin{array}{cc} N & \begin{array}{cc} \begin{array}{c} c\,1\\ \vdots \\ c\,n \end{array} & \begin{array}{c} v\,1\\ \vdots \\ v\,n \end{array} \end{array} \end{array}\right]=\left[\begin{array}{c} R\,1\\ \vdots \\ R\,n \end{array}\right]$$

In Equation (1), *R* represented the *n*-dimensional material element (the influencing factor of enterprise technical management cost), and *R1* and *Rn* were the divisors of *R*. Then, the matter-element matrix of the classical domain (Dai et al., 2018) was defined as follows.

(2) $$R_{j}=\left(N_{j},c,v\right)=\left[\begin{array}{cc} N_{j} & \begin{array}{c} c\,1\,\left(a_{j\,1},b_{j\,1}\right)\\ \vdots \\ c\,n\,\left(a_{j\,n},b_{j\,n}\right) \end{array} \end{array}\right]$$

In Equation (2), *N*_*j*_ represented the grade *j* of the evaluation of factors affecting the technical management cost of enterprise, *cn* represented different characteristics, and *v*_*j*_ = (*a*_1_,*b*_1_) was the range of *cn*. The nodal matter-element matrix was composed of the characteristics that can be transformed into classical matter-element things and the magnitude value range of the corresponding supporting maniac, which can be expressed as follows:

(3) $$R_{p}=\left[\begin{array}{cc} N_{j} & \begin{array}{c} c\,1\,\left(a_{p\,1},b_{p\,1}\right)\\ \vdots \\ c\,n\,\left(a_{p\,n},b_{p\,n}\right) \end{array} \end{array}\right]$$

In Equation (3), *N* was the thing element that had not been evaluated, *cn* represented the *n*-th feature of the element that had not been evaluated, (*a*_*nj*_,*b*_*nj*_) represented the leftmost and rightmost ends of the value range of *cn*, and *b*_*pn*_ represented the maximum value of the range. Then, the linear correlation function (Shen et al., 2019) was selected, which can be expressed as follows:

(4) KjXi={−D(vi,uji)|uji|,vi∈uji−D(vi,uji)D(vi,upi)−D(vi,uji),vi∉uji,

(5) $$\left|u_{j\,i}\right|=\left(b_{j\,i}-a_{j\,i}\right),j=1,2,\cdots ,m;i=1,2\,\cdots ,n,$$

(6) $$D\,\left(v_{i},u_{j\,i}\right)=\left|v_{i}-\left(a_{j\,i}+b_{j\,i}\right)/2\right|-\left(b_{j\,i}-a_{j\,i}\right)/2,$$

(7) $$D\,\left(v_{i},u_{p\,i}\right)=\left|v_{i}-\left(a_{p\,i}+b_{p\,i}\right)/2\right|-\left(b_{p\,i}-a_{p\,i}\right)/2,$$

In Equations (4)–(7), *D*(*v*_*i*_,*u*_*pi*_) represented the moment between the index value and the node field, *D*(*v*_*i*_,*u*_*ji*_) represented the moment between the index value and the classical domain, *v*_*i*_ represented the actual value of the matter element that had not been evaluated, *u*_*ji*_ represented the value interval of the classical domain value, and *u*_*pi*_ represented the value interval of the magnitude value of the node field. Then, the comprehensive correlation degree of *K*_*j*_*X*_*i*_ at grade *j* of the characteristic index of item *i* was calculated, which can be expressed as follows:

(8) $$K_{i}\,\left(P_{O}\right)=\sum_{i=1}^{n}W_{i}\,K_{j}\,\left(X_{i}\right)$$

In Equation (8), *W*_*i*_ was the weight of the characteristic of item *i* and *K*_*i*_(*P*_*O*_) represented the units that had not been evaluated. When *K*_*i*_(*P*_*O*_) was between 0 and 1, the higher the value, the better the conformity; when *K*_*i*_(*P*_*O*_) was between 0 and −1, the smaller the value, the better the conformity; and when *K*_*i*_(*P*_*O*_) was less than −1, it meant that the conditions of the transformed object were not fulfilled.

### Measurement Tools

#### Scale on Sources of Occupational Stress

The scale on sources of occupational stress was compiled referring to the Likert scale (Zhang and Yue, 2017), based on the actual situation of employees in construction enterprises. The original version of the occupational stress scale included six dimensions (i.e., work characteristics, role management, interpersonal relationship, organizational structure and atmosphere, career development, and balance between work and family). The compiled scale on sources of occupational stress also involved six dimensions, namely, work characteristics, stress of role orientation, interpersonal relationship, organizational structure and atmosphere, career development, and balance between work and family, with a total of 37 measurement items. The Likert 5-level scoring system was adopted, and 1–5 points indicated completely non-conforming, not quite conforming, generally conforming, relatively conforming, and completely conforming, respectively. The dimension with a higher score had a greater impact on the stress of the individual. According to the reliability and validity analysis, the internal consistency reliability of the scale with work characteristics, stress of role orientation, organizational structure and atmosphere, career development, interpersonal relationship, and balance between work and family was 0.826, 0.871, 0.905, 0.844, 0.81, and 0.937, respectively.

#### Scale on Stress-Coping Strategies

According to the questionnaire suggested by Hasan and Tumah (2019)’s on stress-coping strategies for employees in general enterprises (Hasan and Tumah, 2019), a new scale on stress-coping strategies was compiled, based on the actual situation of management training in construction enterprises. The scale included two dimensions, namely, the individual and organizational levels, and provided 30 stress-reducing measures. The scoring method adopted the form of “yes” and “no.” The option “yes” counted 1 point and “no” counted 0 points, with the proportion of 30 stress-reducing measures calculated ultimately.

#### Questionnaire to Evaluate Factors Influencing Enterprise Technical Management Cost

The questionnaire adopted in this study included the basic information of the subjects and the evaluation by the subjects on the factors influencing the technical management cost. The hundred-mark scoring system was adopted, in which 1–20 meant very unimportant, 21–40 meant unimportant, 41–60 meant average, 61–80 meant important, and 81–100 meant very important.

### Statistical Analysis

The SPSS19.0 software was used to process the data. The Pearson’s correlation coefficient method was adopted to test the correlation between main variables. The measurement data were expressed as mean ± SD (X ± *s*). The differences in stress sources and stress-coping strategies were analyzed by using a single factor analysis between various demographic variables. Pairwise comparison among six dimensions of stress sources was conducted using group *t*-test.

## Results

### Descriptive Statistics of Stress Sources and Coping Strategies of Employees

**FIGURE 1:**
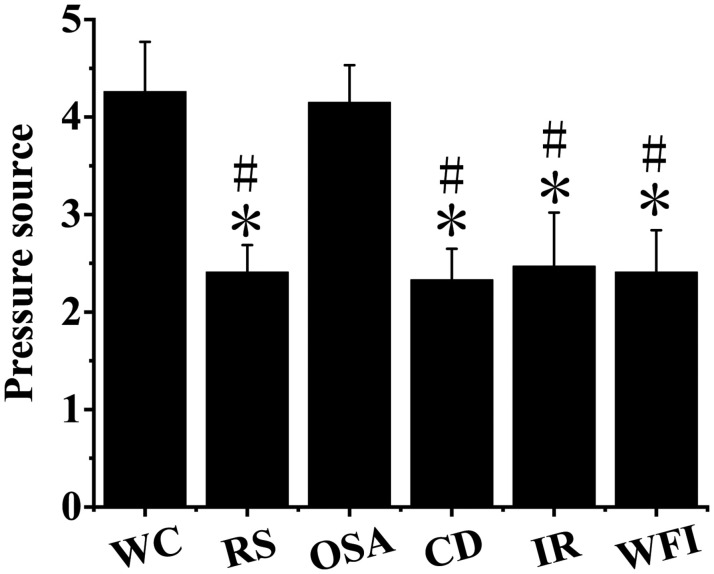
Descriptive statistics of stress sources of employees. ^∗^Indicated that the difference was notable compared with work characteristics (*P <* 0.05). ^#^Indicated that compared with organizational structure and atmosphere, the difference was notable (*P* < 0.05).

As shown in Figure 1, the descriptive statistics on stress-coping strategies and stress sources were carried out, with six dimensions involved, namely, work characteristics, role stress, organizational structure and atmosphere, career development, interpersonal relationship, and balance between work and family. The stress values of work characteristics (4.26 ± 0.511) and organizational structure and atmosphere (4.15 ± 0.382) were higher than those of role stress, career development, interpersonal relationship, and balance between work and family, with notable differences observed (*P <* 0.05). There was no notable difference in stress values between working characteristics and organizational structure and atmosphere stress (*P >* 0.05). The pairwise difference between role stress, career development, interpersonal relationship, and balance between work and family was not notable (*P >* 0.05).

**FIGURE 2:**
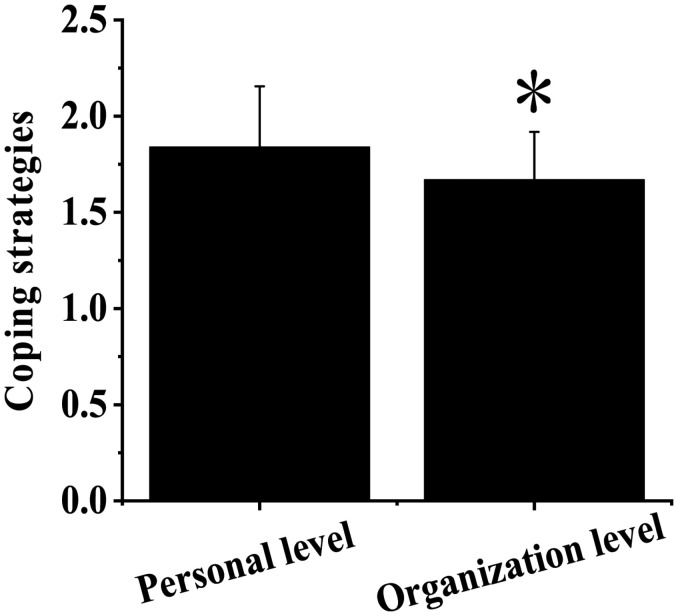
Descriptive statistics of stress-coping strategies of employees. ^∗^Indicated that the difference was notable compared with the individual level (*P* < 0.05).

Figure 2 shows the descriptive statistics of stress-coping strategies of employees. The stress-coping strategies at the individual level scored 1.84 ± 0.315, and the stress-coping strategies at the organizational level scored 1.67 ± 0.248. The stress-coping strategies at the individual level scored higher than at the organizational level, with notable differences observed (*P <* 0.05).

### Difference in Stress Sources Under Various Demographic Variables

As shown in Table 3, for the stress values of work characteristics, there was no notable difference between subjects of different genders and educational backgrounds (*P >* 0.05), while notable differences were observed between subjects of different ages, marital status, and work positions (*P <* 0.05). Among them, the occupational stress of the 30- to 40-year-old employees was higher than that of the other age groups, the occupational stress of the married was higher than that of the unmarried, and the occupational stress of the technical management personnel was higher than that of the ordinary technical personnel.

**TABLE 3:** Differences in stress values of work characteristics under various demographic variables.

Variable	Classification	Work characteristics	χ^2^-value	*P*-value
Gender	Male	4.19 ± 0.428	1.338	0.573
	Female	4.32 ± 0.266		
Age	21–30	3.87 ± 0.362	5.886	0.037
	30–40	4.46 ± 0.164		
	40–51	3.95 ± 0.272		
Marriage status	Married	4.47 ± 0.377	7.175	0.025
	Unmarried	4.05 ± 0.269		
Educational background	Junior college and below	4.26 ± 0.158	2.555	0.069
	Bachelor’s degree	4.17 ± 0.308		
	Master’s degree and above	4.22 ± 0.427		
Work position	General technician	3.97 ± 0.265	6.471	0.017
	Supervisory engineering staff	4.39 ± 0.216		

As shown in Table 4, in terms of the stress values of interpersonal relationship, there were no notable differences in subjects of different genders, marriage status, educational backgrounds, and work positions (*P >* 0.05), while notable differences were observed between subjects of different ages (*P <* 0.05). Among them, the interpersonal stress of employees aged between 30 and 40 years was higher than that of other age groups.

**TABLE 4:** Differences in stress values of interpersonal relationship under various demographic variables.

Variable	Classification	Interpersonal relationship	χ^2^-value	*P*-value
Gender	Male	2.51 ± 0.248	1.429	0.077
	Female	2.44 ± 0.351		
Age	21–30	2.41 ± 0.164	6.528	0.037
	30–40	2.74 ± 0.315		
	40–51	2.35 ± 0.216		
Marriage status	Married	2.55 ± 0.361	1.735	0.183
	Unmarried	2.47 ± 0.551		
Educational background	Junior college and below	2.43 ± 0.207	2.337	0.067
	Bachelor’s degree	2.48 ± 0339		
	Master’s degree and above	2.50 ± 0.272		
Work position	General technician	2.40 ± 0.221	1.667	0.066
	Supervisory engineering staff	2.31 ± 0.153		

As shown in Table 5, in terms of stress values of balance between family and work, there was no notable difference between subjects of different educational backgrounds and work positions (*P >* 0.05), while notable differences were observed between subjects of different genders, ages, and marriage status (*P <* 0.05). Among them, the stress value of males was higher than that of females, the stress value of employees aged 30–40 years was higher than that of employees of other age groups, and the stress value of the married was higher than that of the unmarried.

**TABLE 5:** Differences in stress values of balance between family and work under various demographic variables.

Variable	Classification	Interpersonal relationship	χ^2^-value	*P*-value
Gender	Male	2.65 ± 0.241	5.725	0.017
	Female	2.28 ± 0.165		
Age	21–30	2.21 ± 0.263	5.388	0.029
	30–40	2.69 ± 0.418		
	40–51	2.26 ± 0.176		
Marriage status	Married	2.90 ± 0.253	7.337	0.011
	Unmarried	2.06 ± 0.426		
Educational background	Junior college and below	2.47 ± 0.344	1.957	0.083
	Bachelor’s degree	2.39 ± 0.275		
	Master’s degree and above	2.43 ± 0.319		
Work position	General technician	2.45 ± 0.315	1.528	0.056
	Supervisory engineering staff	2.27 ± 0.462		

### Differences in Stress-Coping Strategies Under Various Demographic Variables

**FIGURE 3:**
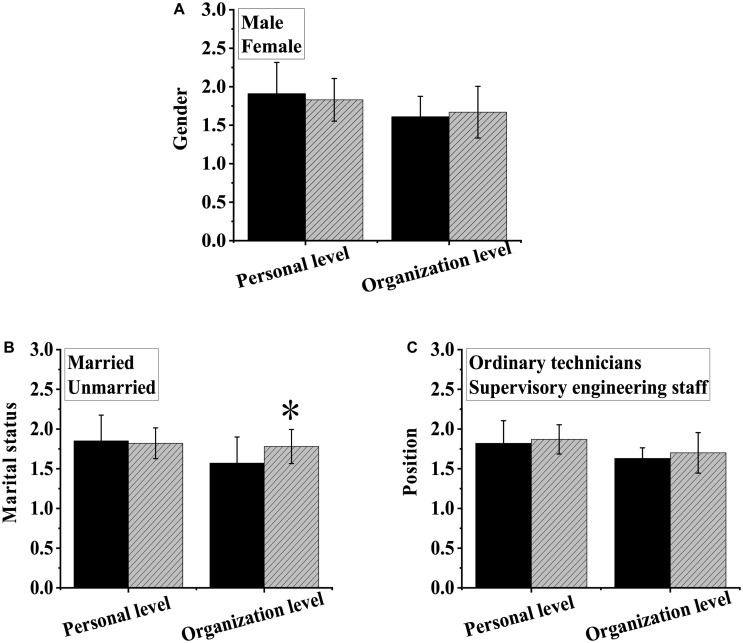
Differences in stress-coping strategies between subjects of different genders **(A)**, marriage status **(B)**, and work positions **(C)**. ^∗^Indicated that compared with married employees, the difference was notable (*P* < 0.05).

As shown in Figure 3, there was no notable difference in stress-coping strategies at both individual and organizational levels between subjects of different genders or work positions (*P >* 0.05). There was no significant difference in stress-coping strategies at the individual level between the married and the unmarried (*P >* 0.05), but notable differences were observed in stress-coping strategies at the organizational level between the married and the unmarried, among which the stress-coping-strategies of the unmarried were significantly higher than those of the married (*P <* 0.05).

**FIGURE 4:**
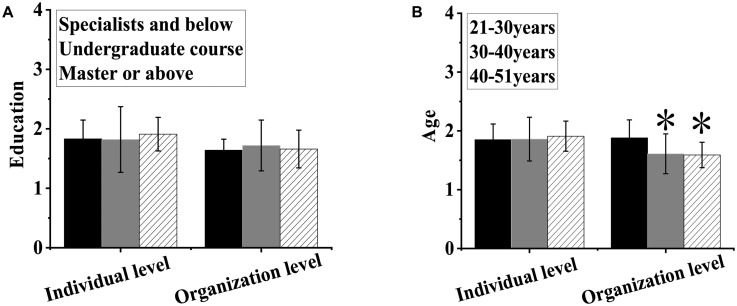
Differences in coping strategies under stress between subjects of different educational backgrounds **(A)** and ages **(B)**. ^∗^Indicated that compared with those between 21 and 30 years old, the difference was notable (*P* < 0.05).

As shown in Figure 4, there was no notable difference in stress-coping strategies at both the individual and organizational levels between subjects of different educational backgrounds (*P >* 0.05). There was no notable difference in stress-coping strategies at the individual level between subjects of different ages (*P >* 0.05), while notable differences were observed in stress-coping strategies at the organizational level between subjects of different ages, among which the stress-coping strategies of employees aged 21–30 years were higher than those of employees of other age groups (*P <* 0.05).

### Expert Score of Factors Influencing Technical Management Cost

Table 6 shows the average scores by the expert, concerning the factors influencing the technical management cost of enterprise. The scores of mechanization and artificial intelligence development, technical equipment rate, and technical management informatization were all higher than 80 points. The number of construction enterprises, the labor population, enterprise scale, corporate culture, and the structure of employees all scored lower than 60 points.

**TABLE 6:** The enterprise technical management cost scored by the expert.

Number	Influencing factor	Average score
1	Location of the enterprise	67.26
2	Enterprise scale	57.29
3	Price index	71.33
4	Development cost ratio	74.71
5	Fixed investment proportion of enterprises	69.40
6	Enterprise labor productivity	60.17
7	Power equipment rate	72.33
8	Contract mode	63.25
9	Mechanization and artificial intelligence development	82.37
10	The number of construction enterprises	51.66
11	Labor cost ratio	73.28
12	Technical equipment rate	86.62
13	Economic globalization	65.88
14	The labor population	57.39
15	Enterprise output value	76.28
16	Employee structure	54.33
17	Profit margin of construction industry	67.43
18	Technical management informatization	89.62
19	Corporate culture	48.39
20	National policy	72.61

### Matter-Element Analysis and Evaluation Model Used to Calculate the Correlation Degree of Each Index

Table 7 shows the correlation degree of influence indicators calculated by the matter-element analysis–based evaluation model. It was noted that the management factors, corporate factors, corporate structure proportions, and insurance and welfare benefits were evaluated as good. Among them, the corporate factors exhibited the greatest impact on the enterprise technology management cost, followed by the management factors and insurance and welfare benefits. Among them, the impact of macro factors was relatively small.

**TABLE 7:** Correlation degree of influence indicators (first-level indicators).

Serial number	Influencing factors	K_*j*(Poor)_	K_*j*(Fair)_	K_*j*(Good)_	K_*j*(Excellent)_	Rating
1	Macro factors	–1.5347	–3.9820	–0.9503	–3.7000	Poor
2	Corporate factors	–2.5700	–1.3309	0.6033	–3.0258	Good
3	Management factors	–2.1324	–0.6800	0.8623	–1.4000	Good
4	Structural proportion	–3.7965	–1.4702	0.8000	–2.7050	Good
5	Insurance and welfare benefits	–0.8193	–0.0332	0.0498	–0.8001	Good

Table 8 shows the correlation degree of each index, calculated by the evaluation model based on the matter-element analysis. Among the factors influencing management cost of Luoyang construction enterprise, some were poorly evaluated, including enterprise scale, enterprise labor productivity, contracting mode, number of construction enterprises, employee structure, and corporate culture; some were evaluated as fair, including location of the enterprise, economic globalization, profitability of the construction industry, and labor population.

**TABLE 8:** Correlation degree of each factor.

Serial number	Influencing factors	K_*j*(Poor)_	K_*j*(Fair)_	K_*j*(Good)_	K_*j*(Excellent)_	Rating
1	Location	0.0400	0.6600	–0.3200	–0.3170	Fair
2	Enterprise scale	0.4810	–0.7100	–0.7562	–0.8255	Poor
3	Price index	–0.6230	–0.3000	0.4000	–0.4619	Good
4	Proportion of development cost	–0.5100	–0.3780	0.4910	–0.5290	Good
5	Proportion of fixed investment	–0.5718	–0.3618	0.5611	–0.5427	Good
6	Labor productivity	–0.1680	0.2711	–0.1729	–0.5271	Poor
7	Power equipment rate	–0.4710	–0.2577	0.5600	–0.5470	Good
8	Contracting mode	0.5100	–0.4670	–0.6759	–0.5710	Poor
9	Mechanical artificial intelligence development	–0.7610	0.2790	–0.2470	–0.4291	Good
10	Number of construction enterprises	0.2500	–0.6100	–0.6710	–0.7380	Poor
11	The proportion of labor cost	–0.5510	–0.3190	0.2700	–0.2164	Good
12	Technical equipment rate	–0.7100	–0.4071	0.5100	–0.2080	Good
13	Economic globalization	–0.0100	0.0710	–0.1820	–0.4170	Fair
14	Number of labor population	–0.2100	0.1600	–0.0600	–0.3700	Fair
15	Output value	–0.6100	–0.3000	0.3100	–0.4700	Good
16	Employee structure	0.0350	–0.0390	–0.4510	–0.5610	Poor
17	Profitability in construction industry	–0.1718	–0.3500	0.2916	–0.2790	Fair
18	Informatization of technology management	–0.5700	–0.4670	0.5100	–0.2710	Good
19	Corporate culture	0.0560	–0.0418	–0.5200	–0.4188	Poor
20	National policy	–0.6510	–0.2788	0.5000	–0.4590	Good

It was evident from Table 9 that some factors were evaluated as good, including the price index, the proportion of development cost, the proportion of fixed investment, the rate of power equipment, the development of mechanical artificial intelligence, the proportion of labor cost, the rate of technical equipment, the output value of enterprise, the informatization of technology management, and the national policy.

**TABLE 9:** Evaluation for each factor.

Serial number	Influencing factors	K_*j*(Poor)_	K_*j*(Fair)_	K_*j*(Good)_	K_*j*(Excellent)_	Rating
1	Price index	–0.6230	–0.3000	0.4000	–0.4619	Good
2	Proportion of development cost	–0.5100	–0.3780	0.4910	–0.5290	Good
3	Proportion of fixed investment	–0.5718	–0.3618	0.5611	–0.5427	Good
4	Power equipment rate	–0.4710	–0.2577	0.5600	–0.5470	Good
5	Mechanical artificial intelligence development	–0.7610	0.2790	–0.2470	–0.4291	Good
6	The proportion of labor cost	–0.5510	–0.3190	0.2700	–0.2164	Good
7	Technical equipment rate	–0.7100	–0.4071	0.5100	–0.2080	Good
8	Output value	–0.6100	–0.3000	0.3100	–0.4700	Good
9	Informatization of technology management	–0.5700	–0.4670	0.5100	–0.2710	Good
10	National policy	–0.6510	–0.2788	0.5000	–0.4590	Good

## Discussion

In the 1980s, the *Time* stated that occupational stress has become a social epidemic. The Workplace Social Report 2019 (hereinafter referred to as the report) released by Maimai, an occupational growth platform in China, reveals that employees generally have low job satisfaction and high occupational stress (Chen, 2019). As a result, it is of utmost importance to educate employees on their mental health and increase the enterprise technology management cost. Based on this, 372 employees of Luoyang construction enterprise were selected as research subjects, to conduct a questionnaire analysis, so as to analyze the differences in stress sources and stress-coping strategies under different demographic variables. The matter-element analysis was used to construct an evaluation model, to comprehensively evaluate the factors influencing the stress of enterprises and the factors influencing technology management cost in construction enterprises. It was found that among the six dimensions, the stress value of work characteristics was the highest, reaching 4.26 ± 0.511, which was in line with the results of suggested by Jeon et al. (2019), indicating that there was much pressure on employees, due to the large workload and the tight deadlines of construction industry. In addition, the stress value of organizational structure and atmosphere ranked the second, reaching 4.15 ± 0.382, suggesting that there were always changes in the actual work, and the enterprise lacked democracy and fairness in decision-making. In the analysis of different demographic variables, it was found that there were no notable differences in work characteristics, role stress, organizational structure and atmosphere, career development, and interpersonal relationship between males and females, but notable differences were observed in the balance between work and family (*P <* 0.05), which was similar to the results of suggested by Scovil et al. (2019), indicating that males had greater stress in balancing family and work than females. There were notable differences in work characteristics, role stress, interpersonal relationship, and balance between work and family between subjects of different ages (*P <* 0.05). The reason may be that the older employees were generally technical elites and management backbone, and assumed more responsibilities. As a result, they felt more stress than the younger employees (Li Q. et al., 2019). No notable differences were observed in the six dimensions between subjects of different educational backgrounds, which indicated that the difficulty in work of enterprise was relatively reasonable. There were notable differences in work characteristics and balance between work and family between the married and the unmarried (*P <* 0.05), which may be because married employees needed to spend more energy and time on the family, and their engagement in work would be relatively reduced, resulting in an increased stress (Wu et al., 2019). There were notable differences in role stress and work characteristics between subjects in different positions (*P* < 0.05), which may be attributed to that the work of ordinary technical personnel was relatively simple, while the technical management personnel needed to assume the main responsibility in enterprise projects, thereby having more occupational stress.

In terms of stress-coping strategies, there was no notable difference in stress-coping strategies at the individual and organizational levels between subjects in different positions and of different genders (*P <* 0.05), suggesting that the stress-coping strategies of male and female employees were basically the same, and the general technical personnel and technical management personnel had similar job responsibilities. There was no notable difference in stress-coping strategies at the individual level between subjects of different ages (*P >* 0.05), while notable differences were observed in stress-coping strategies at the organizational level, and the stress-coping strategies of younger (21–30 years old) employees were higher than those of other age groups (*P <* 0.05), which was basically consistent with the results suggested by Li Y. et al. (2019). It may be because younger employees lacked work experience and reasonable stress-coping strategies, and they needed to rely on stress-coping strategies at the organizational level. There was no notable difference in the stress-coping strategies at both the individual and organizational levels between subjects of different educational backgrounds (*P <* 0.05). The reason may be that the stress-coping strategies at the organizational level were universal and suitable for employees of all educational levels. There was no notable difference in stress-coping strategies at the individual level between the married and the unmarried (*P >* 0.05), but notable differences were observed in stress-coping strategies at the organizational level between subjects of different ages, and the unmarried had higher stress-coping strategies than the married (*P <* 0.05), which was different from the reports suggested by Seyedmohammadi et al. (2019). It may be because the unmarried were more dependent on organization when coping with stress.

In addition, the matter-element analysis was used to construct an evaluation model, to explore the factors that influence the technology management cost of enterprises, with targeted recommendations proposed. In the study, some factors were evaluated as good, including the price index, the proportion of development cost, the proportion of fixed investment, the rate of power equipment, the development of mechanical artificial intelligence, the proportion of labor cost, the rate of technical equipment, the output value of enterprise, the informatization of technology management, and the national policy, which should be regulated and controlled in practice in order to avoid related risks (Liu et al., 2019). However, some limitations should be noted regarding the questionnaire survey. During the survey, if some respondents have a perfunctory attitude toward answering the questions, it will affect the objectivity of the results. Furthermore, the included information and variables are not comprehensive, which reduces the diversity, reliability, and accuracy of the results. The literature research method and interview method were used to screen the factors influencing the technology management cost, and the limitations may be related to the quantity and quality of the literature. As the computer technology develops continuously, artificial intelligence greatly influences enterprise technology management, manifested as improving the information processing capacity, and indirectly influencing the proportion of labor cost, informatization of technology management, and technology equipment rate (Gu et al., 2019). Besides, the national policies are closely related to the development of enterprises, and understanding and actively responding to the national policies will positively affect the enterprise technology management cost.

## Conclusion

The questionnaire survey and the matter-element analysis were used to analyze the stress sources and stress-coping strategies of employees in construction enterprises. Simultaneously, the factors influencing the enterprise technical management costs were explored and the targeted strategies were provided. However, some limitations should be noted in this study. The literature identification method and interview method were used to select the factors, and the small number and poor quality of references reduce the power of the results. Hence, more reliable analysis methods should be explored to improve the reliability of the study. In summary, work characteristics and organizational structure and atmosphere were the two major sources of stress for employees in Luoyang Construction Engineering Group Co., Ltd. Besides, many employees adopted the stress-coping strategies at the individual level, and enterprises needed to strengthen the psychological health education for employees at the organizational level. The factors influencing the technology management cost of enterprises included price index, development cost, fixed investment proportion, power equipment rate, mechanical artificial intelligence, labor cost, rate of technical equipment, the output value, informatization of technology management, and national policy, which enterprises needed to control in practice.

## Data Availability Statement

The raw data supporting the conclusions of this article will be made available by the authors, without undue reservation.

## Ethics Statement

The studies involving human participants were reviewed and approved by the Henan University of Science and Technology, University Ethics Committee. The patients/participants provided their written informed consent to participate in this study. Written informed consent was obtained from the individuals for the publication of any potentially identifiable images or data included in this study.

## Author Contributions

All authors listed have made a substantial, direct and intellectual contribution to the work, and approved it for publication.

## Conflict of Interest

The authors declare that the research was conducted in the absence of any commercial or financial relationships that could be construed as a potential conflict of interest.

## References

[B1] Chen M. (2019). The impact of expatriates’ cross-cultural adjustment on work stress and job involvement in the high-tech industry. *Front. Psychol*. 10:2228. 10.3389/fpsyg.2019.02228 31649581 PMC6794360

[B2] Dai X. S. Fang J. H. Jiang L. F. Xiong Y. Zhang M. F. Zhu S. N. (2018). How does the inclination of the tibial component matter? A three-dimensional finite element analysis of medial mobile-bearing unicompartmental arthroplasty. *Knee* 25 434–444. 10.1016/j.knee.2018.02.004 29685499

[B3] Davis C. H. Comeau J. (2020). Enterprise integration in business education: design and outcomes of a capstone ERP-based undergraduate e-business management course. *J. Inf. Syst. Educ.* 15 287–300.

[B4] Dohi T. Kasai T. Endo H. Wada H. Yanagisawa N. Nojiri S. (2019). CPAP effects on atherosclerotic plaques in patients with sleep-disordered breathing and coronary artery disease: the ENTERPRISE trial. *J. Cardiol.* 73 89–93. 10.1016/j.jjcc.2018.07.002 30177302

[B5] Gu P. F. Xi W. Ye W. P. Shi J. Zhao J. (2019). Extenics matter-element analysis on dilemma problem in HMI design of nuclear power plant. *Nucl. Eng. Des.* 350 176–181. 10.1016/j.nucengdes.2019.05.014

[B6] Hasan A. A. Tumah H. (2019). The correlation between occupational stress, coping strategies, and the levels of psychological distress among nurses working in mental health hospital in Jordan. *Perspect. Psychiatr. Care* 55 153–160. 10.1111/ppc.12292 29781526

[B7] Jeon Y. H. Casey A. N. Vo K. Rogers K. Poole B. Fethney J. (2019). Associations between clinical indicators of quality and aged-care residents’ needs and consumer and staff satisfaction: the first Australian study. *Aust. Health Rev.* 43 133–141. 10.1071/ah17213 29335089

[B8] Jnr B. A. Majid M. A. Romli A. (2020). A generic study on Green IT/IS practice development in collaborative enterprise: insights from a developing country. *J. Eng. Technol. Manage.* 55:101555. 10.1016/j.jengtecman.2020.101555

[B9] Kanaan R. K. Abumatar G. Hussein A. M. A. Al-Lozi M. (2019). Management information system using blockchain technology in an e-commerce enterprise: a systematic review. *J. Bus. Manage.* 7 216–233. 10.25255/jbm.2019.7.3.216.233

[B10] Lambert L. Dedeurwaerdere T. Nyssens M. Severi E. Brolis O. (2019). Unpacking the organisational diversity within the collaborative economy: the contribution of an analytical framework from social enterprise theory. *Ecol. Econ.* 164:106343. 10.1016/j.ecolecon.2019.05.023

[B11] Li Q. Li T. Chen H. T. Xiang E. W. Ruan W. J. (2019). Executives’ excess compensation, legitimacy, and environmental information disclosure in Chinese heavily polluting companies: the moderating role of media pressure. *Corp. Soc. Responsibil. Environ. Manage.* 26 248–256. 10.1002/csr.1676

[B12] Li Y. Huang Z. Wu Y. J. Wang Z. (2019). Exploring how personality affects privacy control behavior on social networking sites. *Front. Psychol.* 10:1771. 10.3389/fpsyg.2019.01771 31417477 PMC6685389

[B13] Liu W. Li X. S. Zheng W. H. Yao R. C. Zheng J. (2019). Preoperative evaluation of the degree of liver fibrosis based on matter-element analysis using serological indicators in patients with hepatocellular carcinoma. *Biosci. Trends* 13 70–76. 10.5582/bst.2018.01311 30867373

[B14] Mayer N. Aubert J. Grandry E. Feltus C. Goettelmann E. Wieringa R. (2019). An integrated conceptual model for information system security risk management supported by enterprise architecture management. *Softw. Syst. Model.* 18 2285–2312. 10.1007/s10270-018-0661-x

[B15] O’Halloran S. M. Connaire A. D. Harte A. M. Leen S. B. (2020). A global-local fretting analysis methodology and design study for the pressure armour layer of dynamic flexible marine risers. *Tribol. Int.* 142:105967. 10.1016/j.triboint.2019.105967

[B16] Rahman F. McEvoy J. W. Ohkuma T. Marre M. Hamet P. Harrap S. (2019). Effects of blood pressure lowering on clinical outcomes according to baseline blood pressure and cardiovascular risk in patients with type 2 diabetes mellitus: the ADVANCE trial. *Hypertension* 73 1291–1299. 10.1161/hypertensionaha.118.12414 31030606 PMC6506385

[B17] Robinson T. G. Davison W. J. Rothwell P. M. Potter J. F. (2019). Randomised controlled trial of a calcium channel or angiotensin converting enzyme inhibitor/angiotensin receptor blocker regime to reduce blood pressure variability following ischaemic stroke (CAARBS): a protocol for a feasibility study. *BMJ Open* 9:e025301. 10.1136/bmjopen-2018-025301 PMC6398677 30782930

[B18] Rodríguez R. Molina-Castillo F. J. Svensson G. (2020). The mediating role of organizational complexity between enterprise resource planning and business model innovation. *Ind. Market. Manag.* 84 328–341. 10.1016/j.indmarman.2019.09.007

[B19] Saeidi P. Saeidi S. P. Sofian S. Saeidi S. P. Nilashi M. (2019). The impact of enterprise risk management on competitive advantage by moderating role of information technology. *Comput. Stand. Interfaces* 63 67–82. 10.1016/j.csi.2018.11.009

[B20] Scovil C. Y. Delparte J. J. Walia S. Flett H. M. Guy S. D. Wallace M. (2019). Implementation of pressure injury prevention best practices across 6 Canadian rehabilitation Sites: results from the spinal cord injury knowledge Mobilization Network. *Arch. Phys. Med. Rehabil.* 100 327–335. 30419231 10.1016/j.apmr.2018.07.444

[B21] Sekhon M. S. Gooderham P. Menon D. K. Brasher P. M. A. Foster D. Cardim D. (2019). The burden of brain hypoxia and optimal mean arterial pressure in patients with hypoxic ischemic brain injury after cardiac arrest. *Crit. Care Med.* 47 960–969. 10.1097/ccm.0000000000003745 30889022

[B22] Seyedmohammadi J. Sarmadian F. Jafarzadeh A. A. McDowell R. W. (2019). Development of a model using matter element, AHP and GIS techniques to assess the suitability of land for agriculture. *Geoderma* 352 80–95. 10.1016/j.geoderma.2019.05.046

[B23] Shen C. Yang Y. He P. Wu Y. (2019). How does abusive supervision restrict employees’ Feedback-seeking Behavior? *J. Manag. Psychol*. 34 546–559. 10.1108/jmp-10-2018-0480

[B24] Song Z. Y. Zhang J. Xiao X. L. Niu D. X. (2019). Multi-energy combined peak dispatching system synthetic benefit evaluation based on variable weight theory and matter-element extension model. *Int. J. Energy Sect. Manag.* 13 713–725. 10.1108/ijesm-08-2018-0004

[B25] Tribst J. P. M. Dal Piva A. M. D. O. Borges A. L. S. Rodrigues V. A. Bottino M. A. Kleverlaan C. J. (2020). Does the prosthesis weight matter? 3D finite element analysis of a fixed implant-supported prosthesis at different weights and implant numbers. *J. Adv. Prosthodont.* 12 67–74. 10.4047/jap.2020.12.2.67 32377319 PMC7183854

[B26] Waters C. M. McDonald S. E. Reseigh J. Grant R. Burnside D. G. (2020). Insights on the relationship between total grazing pressure management and sustainable land management: key indicators to verify impacts. *Rangeland J.* 41 535–556. 10.1071/rj19078

[B27] Watson M. K. Chinnadurai S. K. (2019). Evaluation of noninvasive oscillometric blood pressure monitoring in anesthetized bennett’s wallabies (*macropus rufogriseus*). *J. Zoo Wildlife Med.* 50 389–395. 10.1638/2018-0099 31260205

[B28] Wu W. Wang H. Zheng C. Wu Y. J. (2019). Effect of Narcissism, Psychopathy, and Machiavellianism on Entrepreneurial Intention—The Mediating of Entrepreneurial Self-Efficacy. *Front. Psychol.* 10:360. 10.3389/fpsyg.2019.00360 30846958 PMC6393355

[B29] Zabukovšek S. S. Bharadwaj S. S. Bobek S. Štrukelj T. (2019). Technology acceptance model-based research on differences of enterprise resources planning systems use in India and the European Union. *Eng. Econ.* 30 326–338. 10.5755/j01.ee.30.3.21211

[B30] Zhang X. Yue J. J. (2017). Measurement model and its application of enterprise innovation capability based on matter element extension theory. *Procedia Eng.* 174 275–280. 10.1016/j.proeng.2017.01.136

